# The Use of a Self-triage Tool to Predict COVID-19 Cases and Hospitalizations in the State of Georgia

**DOI:** 10.5811/westjem.2022.4.55001

**Published:** 2022-06-29

**Authors:** Yi-Ting Hana Lee, Mengyu Di, Justin D. Schrager, Zack Buckareff, Rachel E. Patzer, Anna Q. Yaffee

**Affiliations:** * Emory University School of Medicine, Health Services Research Center, Atlanta, Georgia; † Emory University School of Medicine, Department of Surgery, Division of Transplantation, Atlanta, Georgia; ‡ Emory University School of Medicine, Department of Emergency Medicine, Atlanta, Georgia; ¶ Rollins School of Public Health, Department of Epidemiology, Atlanta, Georgia; § Vital Software, Inc., Atlanta, Georgia

## Abstract

**Introduction:**

The coronavirus 2019 (COVID-19) pandemic has created significant burden on healthcare systems throughout the world. Syndromic surveillance, which collects real-time data based on a range of symptoms rather than laboratory diagnoses, can help provide timely information in emergency response. We examined the effectiveness of a web-based COVID-19 symptom checking tool (C19Check) in the state of Georgia (GA) in predicting COVID-19 cases and hospitalizations.

**Methods:**

We analyzed C19Check use data, COVID-19 cases, and hospitalizations from April 22–November 28, 2020. Cases and hospitalizations in GA were extracted from the Georgia Department of Public Health data repository. We used the Granger causality test to assess whether including C19Check data can improve predictions compared to using previous COVID-19 cases and hospitalizations data alone. Vector autoregression (VAR) models were fitted to forecast cases and hospitalizations from November 29 – December 12, 2020. We calculated mean absolute percentage error to estimate the errors in forecast of cases and hospitalizations.

**Results:**

There were 25,861 C19Check uses in GA from April 22–November 28, 2020. Time-lags tested in Granger causality test for cases (6–8 days) and hospitalizations (10–12 days) were significant (P= <0.05); the mean absolute percentage error of fitted VAR models were 39.63% and 15.86%, respectively.

**Conclusion:**

The C19Check tool was able to help predict COVID-19 cases and related hospitalizations in GA. In settings where laboratory tests are limited, a real-time, symptom-based assessment tool can provide timely and inexpensive data for syndromic surveillance to guide pandemic response. Findings from this study demonstrate that online symptom-checking tools can be a source of data for syndromic surveillance, and the data may help improve predictions of cases and hospitalizations.

## INTRODUCTION

The coronavirus 2019 (COVID-19) pandemic has created significant burden on healthcare systems throughout the world.^1^ Syndromic surveillance has been used along with traditional disease surveillance to identify potential outbreaks by using automated data systems to detect early threats.^2^–^4^ The system can collect real-time data based on a range of symptoms rather than laboratory diagnoses, increasing the ability to provide timely information in emergency response.^4^ Early in the pandemic, several countries used syndromic surveillance by monitoring telehealth calls and suspected COVID-19 cases presenting to care.^4^ Studies in Europe and Asia have found that self-reported symptoms collected through mobile applications had strong spatial correlations with confirmed COVID-19 cases^5^ and that by collecting data before and after COVID-19 restrictions, the tool was effective in evaluating control measures.^6^

In April 2020, C19Check.com (C19Check) was launched by Emory University and Vital Software Inc. in Atlanta, Georgia (GA). The online symptom tracker, freely available in 31 languages, prompts users to report their symptoms and then generates evidence-based summaries of risk of COVID-19 infection and advice for seeking healthcare. We sought to examine the usefulness of C19Check as a syndromic surveillance tool in GA by assessing whether C19Check use can predict COVID-19 cases and hospitalizations. The findings have important implications on novel methods for syndromic surveillance during current and future pandemics.

## METHODS

We analyzed C19Check use by location from April 22–November 28, 2020. Daily incident COVID-19 cases and hospitalizations in GA over the same period were extracted from the Georgia Department of Public Health (GDPH) data repository. COVID-19 cases were identified through positive molecular and antigen tests. Hospitalizations were based on confirmed cases hospitalized at the time the case was reported to GDPH or interviewed. C19Check use was defined as the number of online forms completed per day. We excluded from the analysis users who reported a ZIP code outside of GA.

To examine whether C19Check use can predict COVID-19 cases and hospitalizations, we conducted a Granger causality test^7^ for cases and hospitalizations separately with C19Check use. First, we took the log of cases/hospitalizations and C19Check use and applied a unit root test to determine data stationarity.^8^ For hospitalizations, the data was not stationary; so we applied the first difference and conducted the Johansen co-integration test to assess stationarity. Vector autoregression models (VAR) were fitted with different time-lags, and the time-lag with minimum Akaike information criterion for best fit was selected (seven days for cases and 11 days for hospitalizations).

We conducted hypothesis testing with a Granger causality test to determine whether including C19Check use can better predict cases and hospitalizations separately than including cases or hospitalizations data alone in the models, respectively. Time-lags of 6–8 days (cases) and 10–12 days (hospitalizations) were selected for the test to account for time-lag sensitivity in the Granger causality test. Following hypothesis testing, separate VAR models were fitted to predict cases and hospitalizations in GA from November 29–December 12, 2020. We calculated mean absolute percentage error (MAPE) to estimate the errors in forecast of cases and hospitalizations. All analyses and plots were created in R version 4.1.0 (RStudio; Boston, MA). The study was reviewed by the institutional review board (IRB), which determined that IRB review and oversight was not required as the project did not meet the criteria for human subjects’ research.

## RESULTS

From April 22–November 28, 2020, there were 85,996 total C19Check uses, of which 25,861 uses were in GA. During the study period, the peak C19Check uses were on July 7 and November 20. The highest daily COVID-19 cases were on November 21 and July 24, while the highest daily hospitalizations were on July 13 and 10, 2020.

**Figure 1:**
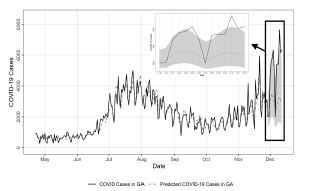
Epidemic curve of measured daily COVID-19 incident cases and C19Check-predicted COVID-19 cases from April 22–December 12, 2020 in the state of Georgia. The upper and lower confidence intervals (gray shading) from the fitted VAR model with a time-lag of seven days are depicted from November 29–December 12, 2020.

All time-lags for C19Check use and COVID-19 cases were significant (*P*=< 0.05) in the Granger causality test. This indicates that information on C19Check use six to eight days prior to a specific date between April 22–November 28, 2020 was better able to predict COVID-19 cases on that date than using the prior information on COVID-19 cases alone. In the VAR model fitted with a time-lag of seven days, we predicted cases from November 29–December 12, 2020 and found that MAPE was 39.63% (Figure 1).

**Figure 2:**
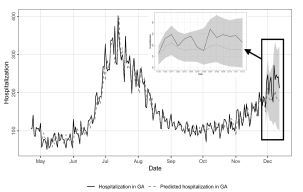
Epidemic curve of measured daily incident COVID-19-related hospitalizations and C19Check-predicted COVID-19 related hospitalizations from April 22–December 12, 2020 in the state of Georgia. The upper and lower confidence intervals (gray shading) from the fitted VAR model with a time-lag of 11 days are depicted from November 29–December 12, 2020.

For COVID-19 hospitalizations, results were significant for all time lags (*P* <0.05) in the Granger causality test. We found that including information on C19Check use in the model was more useful in predicting hospitalizations than including information on hospitalizations alone. In the VAR model fitted with a time-lag of 11 days, the MAPE for predicted hospitalizations was 15.86% (Figure 2).

## DISCUSSION

By including C19Check use in the Granger causality test, we increased our ability to predict daily COVID-19 cases and hospitalizations compared to using information on cases and hospitalizations alone. In the fitted VAR models, the MAPE for cases and hospitalizations predictions were 39.63% and 15.86%, respectively. The amount of error in the forecast is likely because C19Check use itself is not a cause of the surges and declines in cases and hospitalizations. The significant results for all time-lags tested indicate that our findings on the predictability of C19Check use were not impacted by time-lags, demonstrating the effectiveness of C19Check as a tool for syndromic surveillance of COVID-19 cases and hospitalizations in GA. Other real-time syndromic surveillance tools have been used to detect early signals, monitor population transmission dynamics and identify hotspots in different countries^5^,^6^,^9^,^10^ and various regions of the US.^9^,^11^ However, we also evaluated the performance of an internet-based self-triage tool in predicting COVID-19 cases and hospitalizations.

## LIMITATIONS

There are several limitations to our study. First, since COVID-19 is a novel virus, the reporting of cases and hospitalizations changed throughout the pandemic. Testing capacity was limited early on, and case counts were likely underestimated. The reporting of hospitalization data from GDPH did not reflect those currently hospitalized with COVID-19 and likely underestimated the actual total number of hospitalizations. Second, the use of C19Check requires volitional community participation and its users may not be generalizable to the overall GA population. Third, as the pandemic progressed in the winter of 2020–2021, we observed reduced C19Check use despite the surges in COVID-19 cases, hospitalizations, and deaths as well as the presence of variants. This may be due to a combination of factors including the increase of available predictor tools, “pandemic fatigue” leading to relaxation of precautions,^12^ and a better general understanding of COVID-19 symptoms with less reliance on web-based tools. However, C19Check was able to predict cases and hospitalizations despite these limitations. A major strength of our study is the availability of data beyond the initial months of the pandemic, which allowed us to examine the effectiveness of C19Check to predict cases and hospitalizations during surges and declines.

## CONCLUSION

The use of self-triage tools such as C19Check can help predict cases and hospitalizations during the pandemic. In settings where laboratory tests are limited, contact tracing is challenged and public health responses are hindered by the lack of information on incident cases, infection rates, and transmission dynamics,^13^ a real-time, symptom-based assessment tool can provide timely and inexpensive data for syndromic surveillance in order to guide pandemic response.^13^ Further research is warranted to understand the factors influencing predictability and evaluate the impact of the tool on healthcare systems. C19Check and other interactive web-based platforms have highlighted an opportunity for self-triage tools to predict trends and guide public health measures in pandemic response.

## Supplementary Information
